# Genitopatellar Syndrome With a Novel Variant in the KAT6B Gene: Supporting Spectrum Delineation

**DOI:** 10.7759/cureus.69989

**Published:** 2024-09-23

**Authors:** Warren Back, Adam Mierzwa, Naeem Mahfooz

**Affiliations:** 1 Surgery/Neurosurgery, The University of Toledo, College of Medicine and Life Sciences, Toledo, USA; 2 Neurology, The University of Toledo, Toledo, USA; 3 Pediatric Neurology, The University of Toledo, Toledo, USA

**Keywords:** agenesis of the corpus callosum, genitopatellar syndrome, genitourinary complications, gps, kat6b, lysine acetyltransferase, say-barber-biesecker-young-simpson syndrome, sbbyss

## Abstract

Genitopatellar syndrome (GPS) and Say-Barber-Biesecker-Young-Simpson syndrome (SBBYSS) are rare genetic disorders linked to mutations in the Lysine Acetyltransferase 6B (KAT6B) gene, affecting histone acetylation regulation and developmental processes. We present a case of an African American infant with classic GPS features and a novel KAT6B gene mutation (c.4066del, p.Glu1356Argfs*23). The patient exhibited skeletal anomalies, neurologic deficits, and genitourinary abnormalities, consistent with GPS. Genetic analysis revealed a de novo heterozygous pathogenic variant, adding to the growing understanding of KAT6B-related disorders. Reviewing recent literature, we found an increased prevalence of reported cases and novel gene variants, supporting the delineation of GPS and SBBYSS. Furthermore, analysis suggests a preference range within the gene associated with GPS phenotypes, challenging the notion of a spectrum of KAT6B-related disorders. As genetic sequencing advances, continued reporting of cases will inform decisions regarding the classification of these disorders.

## Introduction

Genitopatellar syndrome (GPS) and Say-Barber-Biesecker-Young-Simpson syndrome (SBBYSS) are extremely rare genetic disorders stemming from mutations in the lysine acetyltransferase 6B (KAT6B) gene [1]. KAT6B encodes a member of the MYST family of histone acetyltransferases, and investigations reveal a notable reduction in histone H3 and H4 acetylation levels in cells derived from patients, indicating a direct functional impact of these alleles on histone acetylation regulation. These findings not only elucidate the genetic underpinnings of these disorders but also shed light on the intricate role of histone acetylation regulation in developmental processes [2].

GPS, first described by Cormier-Daire et al. in 2000, is characterized by skeletal anomalies such as absent or hypoplastic patellae, club feet, and flexion contractures, alongside neurologic impairments and genitourinary anomalies [3]. In contrast, SBBYSS manifests with distinct features, including neurologic deficits, ocular anomalies, and skeletal abnormalities, such as long thumbs and great toes, alongside similar genitourinary and facial dysmorphisms observed in GPS. These syndromes present with overlapping clinical features and even an intermediate phenotype in some cases [4].

The overlap of phenotypes, along with many other unique features appearing in both disorders and even KAT6B mutations that present distinctly from GPS and SBBYSS, has led many researchers to call for the renaming of KAT6B-related disorders to include a spectrum instead of a delineation of two syndromes. This call does not come without opposition from others supporting the delineation. Therefore, understanding the genotype-phenotype correlations within KAT6B disorders is crucial in making a decision. Herein, we aim to add to the current body of knowledge by introducing the case of an infant with typical GPS features and a novel mutation of the KAT6B gene.

## Case presentation

An African American male was born full term via a cesarean section to non-consanguineous parents with a maternal history of hypothyroidism and sickle cell trait and no significant paternal history. Pregnancy was complicated by breech presentation, preeclampsia, and multiple fetal anomalies observed on prenatal ultrasound, including agenesis of the corpus callosum, absent coccyx, ambiguous genitalia, anal atresia, dilation of the urinary system, and polyhydramnios.

**Figure 1:**
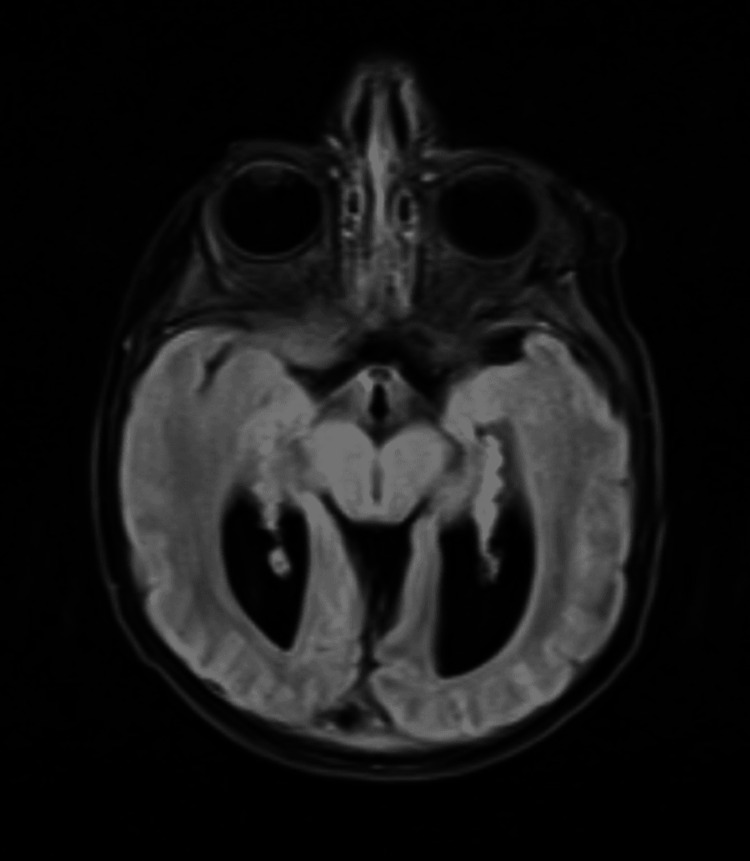
MRI brain (T2 axial) showing colpocephalic configuration of the ventricular system and absence of the corpus callosum (motion artifact present).

**Figure 2:**
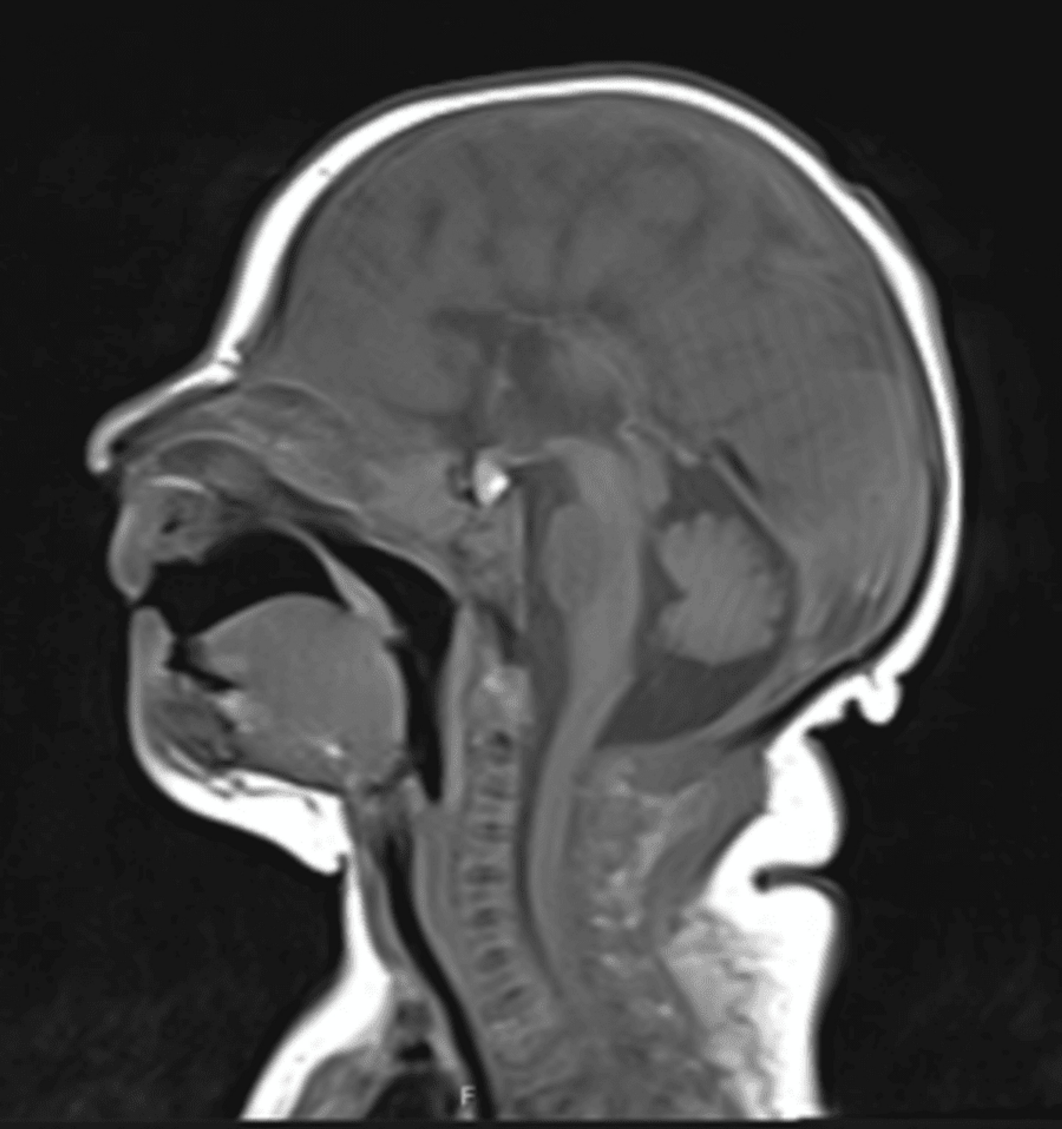
MRI brain (T1 sagittal) showing absence of the corpus callosum (motion artifact present).

**Figure 3:**
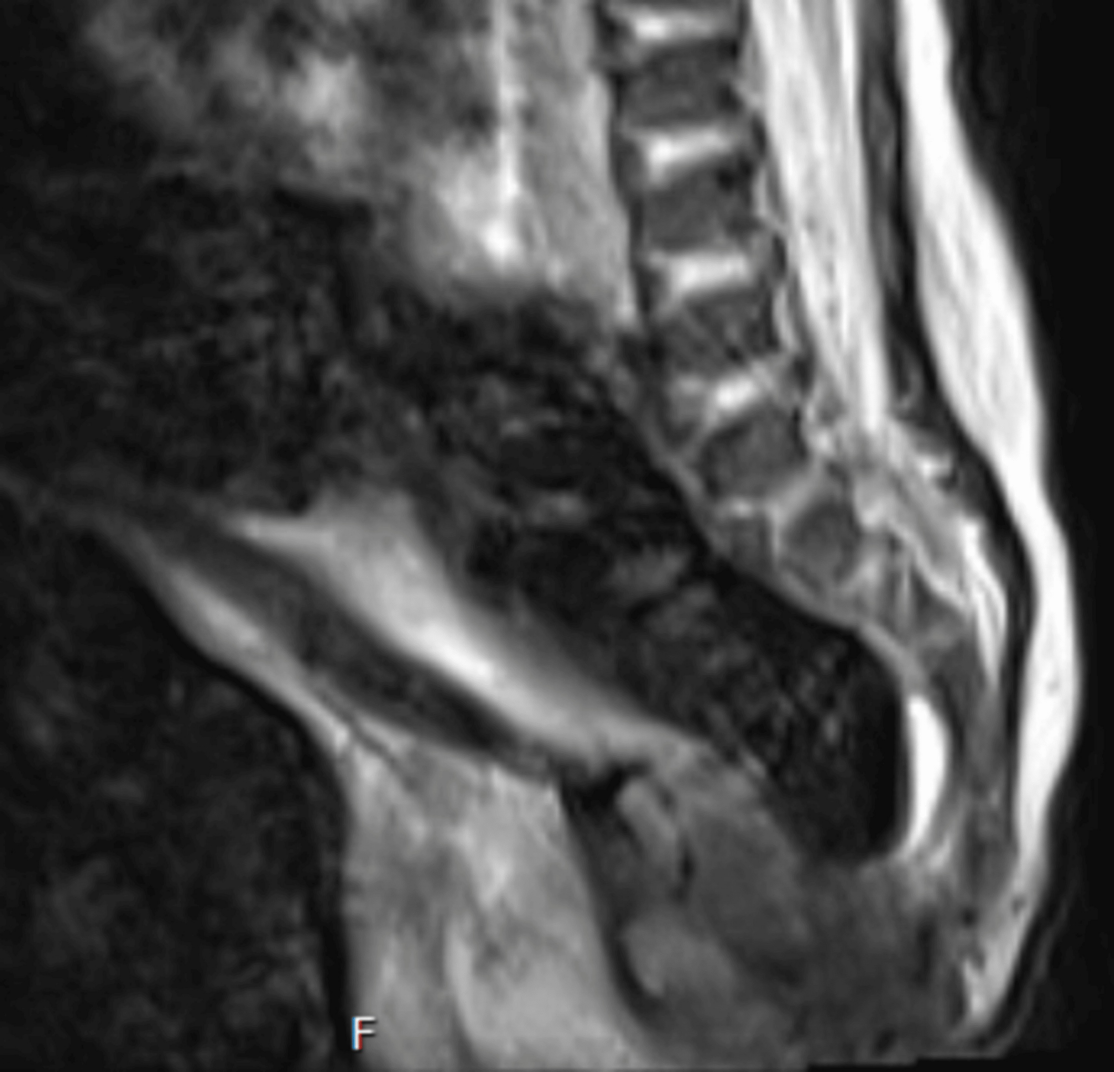
MRI lumbar spine (T2 sagittal) demonstrating sacral agenesis, low-lying conus medullaris, and anal atresia/imperforate anus (motion artifact present).

**Figure 4:**
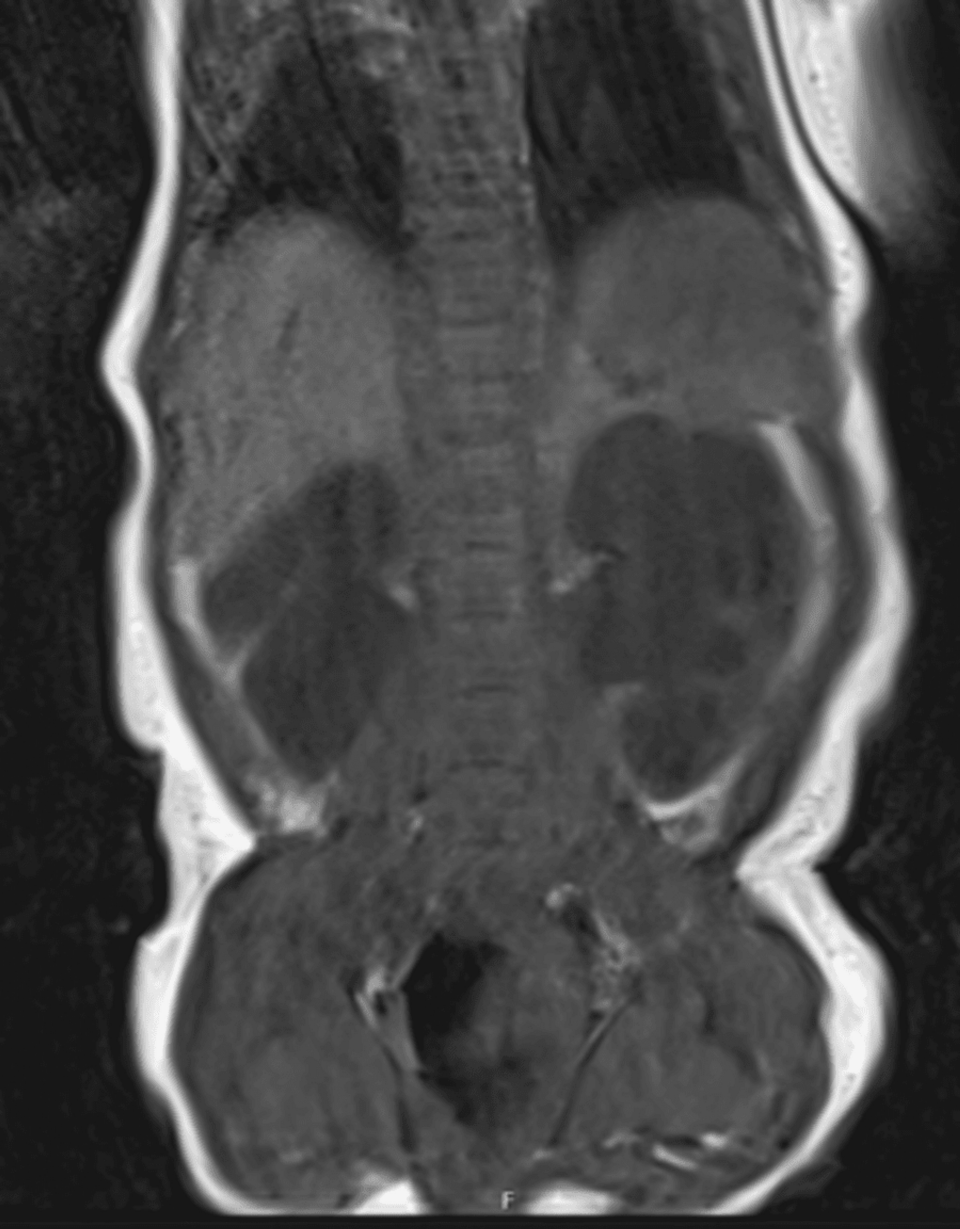
MRI lumbar spine (T1 coronal) showing bilateral hydronephrosis (motion artifact present).

He was born at a healthy birth weight with initial Apgar scores of 8 and 9 at 1 and 5 minutes, respectively. He developed respiratory distress approximately 10 minutes after birth, necessitating CPAP, and subsequently transferred to the NICU. Further evaluation at this time revealed a colpocephalic ventricular system (Figure 1), absent corpus callosum (Figures 1, 2), patent ductus arteriosus, patent foramen ovale, ambiguous genitalia with undescended testicles and absent Mullerian structures, imperforate anus (Figure 3), low-lying conus-medullaris (Figure 3), laryngomalacia, bilateral hydronephrosis (Figure 4), bilateral club feet, dysplasia of the right hip, absent distal phalanges of the left hand, and overriding sutures. Multiple services were consulted regarding these abnormalities.

During his extensive evaluation in the hospital, he underwent genetic analysis through Prevention Genetics. This revealed a de novo heterozygous pathogenic variant of the KAT6B gene: (c.4066del, p.Glu1356Argfs*23); this variant has not yet been mentioned in the current literature per their report. They also suggested that although he displays multiple features of the VACTERL syndrome, this is not a typical presentation and is thus better explained by the genetic mutation.

Surgery to correct the anal atresia and laryngomalacia was performed without complications. He was started on amoxicillin for infection prophylaxis given the hydronephrosis, as well as iron supplementation for a slight anemia. Hormonal analysis was normal. The remainder of his hospital course over the next couple of weeks was unremarkable and consisted of multiple specialist evaluations. He was discharged on day 39 of life with outpatient follow-up scheduled for primary care, cardiology, otorhinolaryngology, genetics, nephrology, neurology, neurosurgery, orthopedics, pediatric surgery, urology, and gastroenterology.

The eight-week-old patient presented with his mother to our outpatient neurology clinic for post-hospital follow-up due to the brain abnormalities previously described. She reported no seizure-like activity or other concerns. Electroencephalograms performed during hospitalization showed no epileptiform activity. On examination, he was interactive, able to track objects with both eyes, and had normal muscle tone in the upper extremities. Lower extremity evaluation was deferred due to bilateral casts for clubfoot treatment. A weak suck and inspiratory stridor were noted on the exam with no evidence of palate deformity. Low-set ears were also appreciated. No intervention was suggested at this time, and follow-up was scheduled for three months to monitor progression.

## Discussion

Our patient has all of the classic major features of GPS, ignoring patellar hypoplasia/agenesis due to age, and the absence of all those of SBBYSS (Table 1). His novel KAT6B gene variant (c.4066del, p.Glu1356Argfs*23) falls within the proximal segment of exon 18, which is the most commonly reported region for mutation and the region with the highest incidence of GPS diagnosis [4,5]. 

**Table 1:** Major clinical features of GPS and SBBYSS GPS: Genitopatellar syndrome; SBBYSS: Say-Barber-Biesecker-Young-Simpson syndrome Classic features of GPS and SBBYSS, with evidence of overlapping phenotypes. Adapted from Lemire et al. [4].

Major features of GPS	Major features of SBBYSS
Genital anomalies	Long thumbs/great toes
Patellar hypoplasia/agenesis	Immobile mask-like face
Contractures at the hips, knees and/or clubfoot	Blepharophimosis and/or ptosis
Agenesis of the corpus callosum	Lacrimal duct anomalies
Renal anomalies (hydronephrosis or multiple renal cysts)	Patellar hypoplasia/agenesis

Lemire et al. reported a prevalence of 89 known cases of KAT6B gene mutation in 2020 [4]. There have since been 12 PubMed-indexed articles, presenting 67 unique patients, bringing the total to 156 [5-16]. The current division of diagnosis is 92 with SBBYSS, 36 with GPS, 23 with intermediate phenotype, five not otherwise specified, and one patient presenting with a phenotype unique to either syndrome. Now, with the addition of our patient, whom we declare to be the 37th known case of GPS, the current known prevalence of a KAT6B gene mutation is 157 patients, consisting of 92 SBBYSS, 37 GPS, 23 intermediate, five not otherwise specified, and one unique presentation. 

Yabumoto et al. reported all currently known variants of the KAT6B gene in 2021 [6]. Since this analysis, four articles have been published on the topic, with multiple novel mutations in the gene [12-15]. There were nine patients reported in these studies, one of which had a mutation in exon 7 (c.1013A>G, p.(Tyr338Cys)), leading to a unique phenotype outside the spectrum of GPS and SBBYSS [12]. The other eight patients were diagnosed with SBBYSS, and 7 of the eight presented novel gene mutations. Therefore, including our patient, nine new variants have been discovered in the KAT6B gene since Yabumoto’s report (Table 2).

**Table 2:** KAT6B variants described since 2021 SBBYSS: Say-Barber-Biesecker-Young-Simpson syndrome; GPS: Genitopatellar syndrome. Data collected from all articles published since Yabumoto's report in 2021 [6,12-15]. These reports taken together provide a comprehensive collection of the data relating to the KAT6B-related disorders as of 2024, including the disorder, position of the genetic variation on the NM_012330.3 reference sequence, exon, and novelty of the reported mutation.

KAT6B related disorder	Position of variant on NM_012330.3 reference sequence	Exon	Novel vs Previously Described	Presenting author
Unique Syndrome	c.1013A>G, p.(Tyr338Cys)	7	Novel	Nishimura et al. [12]
SBBYSS	c.3040C>T (p.Gln1014*)	16	Novel	Chen et al. [13]
SBBYSS	c.3147G > A; p.(Pro1049=) p.Ala1008Argfs*62	16	Previously Described	Klaniewska et al. [14]
SBBYSS	c.3852_3864del; p.(Asp1284Glufs*46)	18	Novel	Klaniewska et al. [14]
SBBYSS	c.4026dup; p.(Pro1343Thrfs*4)	18	Novel	Klaniewska et al. [14]
SBBYSS	c.5819del; p.(Gln1940Argfs*11)	18	Novel	Klaniewska et al. [14]
SBBYSS	c.4455dup; p.(Asn1486*)	18	Novel	Klaniewska et al. [14]
SBBYSS	c.5012del; p.(Gly1671Alafs*44)	18	Novel	Klaniewska et al. [14]
SBBYSS	c.3147G>A; p.P1049P	18	Novel	Darvania et al. [15]
GPS	c.4066del, p.Glu1356Argfs*23	18	Novel	This Report

Zhang et al. conducted a retrospective analysis of semiology among all currently known patients with KAT6B disorders in 2020, shedding light on the spectrum’s complex manifestations. Neurological features, such as agenesis or hypoplasia of the corpus callosum, microcephaly, and hypotonia, were consistently reported, along with developmental delays and intellectual disabilities. Moreover, individuals often exhibit craniofacial anomalies, including blepharophimosis and cleft lip/palate, while genitourinary anomalies like cryptorchidism or clitoromegaly are common. Cardiovascular defects, musculoskeletal anomalies such as patellar abnormalities, and respiratory issues, including tracheomalacia, are also prevalent. Additionally, ocular abnormalities and hearing impairments are frequent, alongside gastrointestinal anomalies like anal atresia and feeding difficulties [5]. These findings support the core aspects first described by Cormier-Daire et al., while also elucidating the variable expressivity that is becoming a more accepted understanding of the KAT6B disorder spectrum.

Other work focusing on the location of the variation within the gene has shown possible relationships between site and phenotype. Vlckova et al. concluded that the mutations in mid-exon 18, representing the C-terminal end of the acidic domain, had more variable expressivity, whereas other regions of the gene were more specific to either GPS or SBBYSS [16]. Likewise, SBBYSS has been recognized to have the highest density of variation in the distal part of exon 18, and GPS is more likely at the distal end of exon 17 and the proximal portion of exon 18 [17,18]. These authors advocate for a delineation in GPS and SBBYSS, opposing the concept of a spectrum or continuum of KAT6B-related disorders.

The known genetic mutations that cause GPS, as shown in the figure reported by Yabumoto et al., typically fall within a narrower amino acid range (1194-1515) when compared to SBBYSS and intermediate phenotypes [6]. This range falls within the distal portion of exon 17 and the proximal portion of exon 18, and crosses between the acidic and Ser/Met protein domains. Our patient’s variant, the only novel GPS variant described in the past couple of years, continues to follow this trend. Although SBBYSS and intermediate phenotypes are demonstrated within this range, their density is higher in other regions of the gene. This is partly supported by the remainder of the novel variants described in Table 2, as 5 of the 8 SBBYSS are outside of this range. The significance of this postulated GPS-preference range is not known but may argue against removing the delineation of KAT6B-related disorders. 

## Conclusions

In conclusion, we identified a novel mutation of the KAT6B gene in an African American infant male with many of the typical GPS features. Additionally, we aggregated all new gene variants described since 2021 and combined them with prior collections. Despite their often distinct clinical presentations, GPS and SBBYSS occasionally have overlapping phenotypes, highlighting the complexity of this genetic region. Lastly, we postulate that there may be a preference range within the gene from which GPS arises, refuting the idea of a KAT6B disorder spectrum. Because of the rare nature of these disorders, these conclusions are limited to a smaller sample size and a lack of large-scale analysis. As genetic sequencing technologies continue to advance and become more accessible, we expect to see a rise in reported cases that may aid in the decision of whether or not to keep the delineation of these disorders.
